# Synaptically evoked glutamate transporter currents in Spinal Dorsal Horn Astrocytes

**DOI:** 10.1186/1744-8069-5-36

**Published:** 2009-07-01

**Authors:** Haijun Zhang, Wenjun Xin, Patrick M Dougherty

**Affiliations:** 1 Department of Anesthesiology and Pain Medicine, University of Texas M. D. Anderson Cancer Center, Houston, TX 77030, USA

## Abstract

**Background:**

Removing and sequestering synaptically released glutamate from the extracellular space is carried out by specific plasma membrane transporters that are primarily located in astrocytes. Glial glutamate transporter function can be monitored by recording the currents that are produced by co-transportation of Na^+ ^ions with the uptake of glutamate. The goal of this study was to characterize glutamate transporter function in astrocytes of the spinal cord dorsal horn in real time by recording synaptically evoked glutamate transporter currents.

**Results:**

Whole-cell patch clamp recordings were obtained from astrocytes in the spinal substantia gelatinosa (SG) area in spinal slices of young adult rats. Glutamate transporter currents were evoked in these cells by electrical stimulation at the spinal dorsal root entry zone in the presence of bicuculline, strychnine, DNQX and D-AP5. Transporter currents were abolished when synaptic transmission was blocked by TTX or Cd^2+^. Pharmacological studies identified two subtypes of glutamate transporters in spinal astrocytes, GLAST and GLT-1. Glutamate transporter currents were graded with stimulus intensity, reaching peak responses at 4 to 5 times activation threshold, but were reduced following low-frequency (0.1 – 1 Hz) repetitive stimulation.

**Conclusion:**

These results suggest that glutamate transporters of spinal astrocytes could be activated by synaptic activation, and recording glutamate transporter currents may provide a means of examining the real time physiological responses of glial cells in spinal sensory processing, sensitization, hyperalgesia and chronic pain.

## Background

Spinal glial cells are now recognized as important participants in mechanisms of sensory encoding and the plasticity underlying the generation of spinal sensitization, hyperalgesia and chronic pain. Nevertheless, studies implicating glial cells in these processes have been confined to anatomical and behavioral approaches. An important facet of glial functions within the central nervous system (CNS) that may be fundamental to their contribution to sensory encoding in the spinal cord is the clearance of glutamate from the extracellular space via specific plasma membrane glutamate transporters [1-5]. Five cell membrane glutamate transporter proteins have been cloned, including EAAT1, EAAT2, EAAT3, EAAT4 and EAAT5 [5,6]. The rodent analogs of EAAT1 (GLAST) and EAAT2 (GLT-1) are expressed primarily in glial cells in the CNS [1,5]. Synaptic transmission in various brain structures such as the hippocampus has been shown to be potently shaped and regulated by the functional status of these transporters [3,7]. Glutamate transporters are also involved in spinal nociceptive processing [8]. For example, inhibition of glutamate transporters in the spinal cord elevates spinal extracellular glutamate concentrations, alters glutamatergic synaptic transmission among lamina II neurons [9], and produces spontaneous nociceptive behaviors [10]. Dorsal horn neurons develop hyper-responsiveness to mechanical and thermal stimuli after blockade of glutamate transporters [11]. Peripheral nerve injury [12,13] or chemotherapy [14] induced down-regulation of glutamate transporter protein expression and attenuation of glutamate uptake activity in the spinal dorsal horn.

The hypothesis tested here is that real time physiological responses of glial cells, specifically astrocytes, to sensory inputs could be measured by analysis of glutamate transporter currents. The synaptic activation of glutamate transporter currents in glial cells has been demonstrated in culture [15,16] and in brain slices [17,18]. So far these currents have not been characterized in spinal glial cells. Here we demonstrate that electrophysiologically characterized spinal astrocytes express two types of glutamate transporters, GLAST and GLT-1. The glutamate transporter currents in spinal astrocytes evoked by single stimulation from dorsal root entry zone share some similar properties with hippocampal astrocytes but also have their unique characteristics.

## Results

### Identification of spinal astrocytes

**Figure 1:**
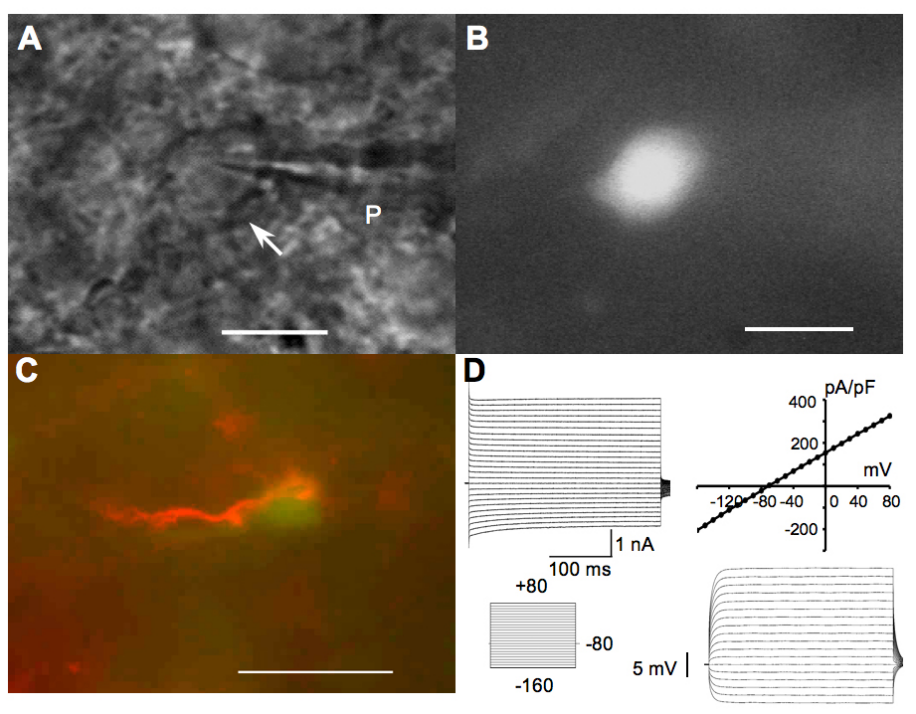
**Identification of astrocytes in spinal dorsal horn**. An astrocyte (arrow) with attached patch pipette (P) is shown when viewed in DIC (A) and under epifluorescence when filled with Lucifer Yellow (B). C, Anti-GFAP antibody labeling (red) superimposed on the cell loaded with Lucifer Yellow (green) shown in B. D, under voltage clamp, inward and outward currents (upper left) were evoked in the same cell, by depolarizing and hyperpolarizing voltage steps from a holding potential of -80 mV (lower left). I/V curve (upper right) shows the nearly linear current-voltage relationship with slight rectification during hyperpolarization from the recorded cell. Whole-cell membrane potential changes (lower right) induced by 200 ms steps of current (-500 to 1200 pA) recorded from the same cell, noticing there is no action potential induced. Scale bar = 10 μm in A and B; 20 μm in C.

Whole-cell recordings were obtained from 45 glial cells in the SG of spinal cord dorsal horn. Glial cells were first visually identified as small, rounded cells with a soma diameter less than 10 μm (Fig. 1A). We further distinguished glial cells from neurons by their electrophysiological properties after obtaining the whole-cell recording configuration. Glial cells were characterized by absence of action potentials to injection of depolarizing currents steps up to 1200 pA while in current clamp mode and by the demonstration of passive current responses to voltage steps while in voltage clamp mode (Fig. 1D). In addition, recorded cells had a characteristically low mean input resistance of 24.4 ± 2.4 MΩ (n = 45), negative resting membrane potential of -74.1 ± 0.5 mV (n = 45), and mean membrane capacitance of 11.4 ± 1.0 pF (n = 45).

Previous studies have shown that the four different types of glial cells in spinal cord gray matter including astrocytes, oligodendrocytes, oligodendrocyte precursors and astrocyte precursors, display distinct membrane current responses to voltage steps [19]. Spinal cord astrocytes were differentiated from oligodendrocytes and precursor cells by their passive, non-decaying inward and outward currents in response to hyperpolarizing and depolarizing voltage steps, respectively. Astrocytes typically showed small rectifying currents with voltage steps below -80 mV. In the present study, only cells showing electrophysiological characteristics of spinal cord astrocytes were chosen, otherwise recordings were discarded. Recorded cells were marked with Lucifer Yellow contained in the pipette solution and the physiological criteria for cell selection were validated post-recording by immunostaining for the astrocyte-specific marker glial fibrillary acidic protein (GFAP) (Fig. 1C).

### Characteristics of synaptically evoked glutamate transporter currents in spinal astrocytes

**Figure 2:**
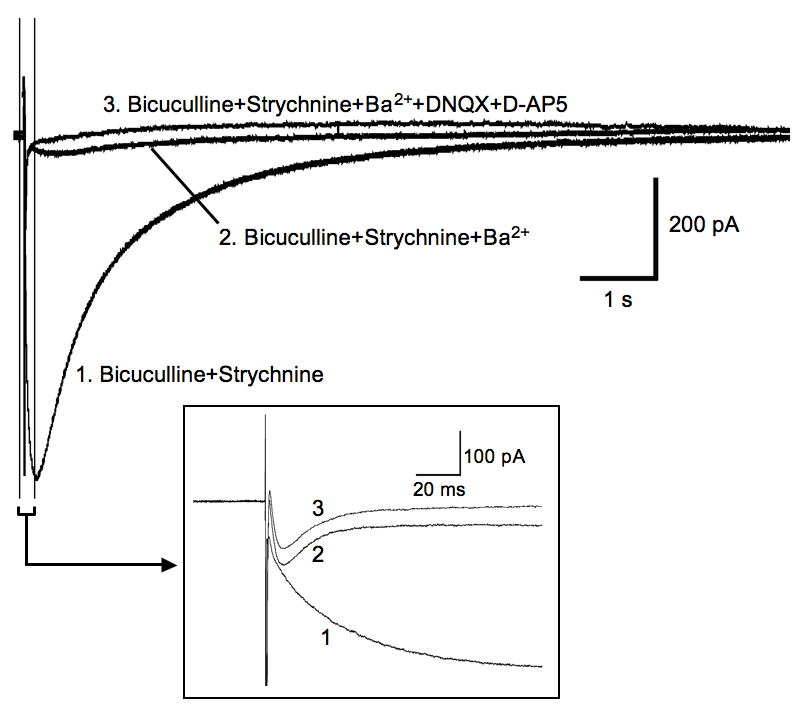
**Spinal dorsal root entry zone stimulation evoked an inward current in spinal astrocytes**. In the presence of antagonists to GABA_A _and glycine receptors (bicuculline and strychnine), a slow-decaying inward current was recorded from a spinal astrocyte holding at -80 mV (n = 15, curve 1). Ba^2+ ^(1 mM) largely blocked the slow-decaying inward current, but unmasked a fast-decaying inward current (curve 2, also visible with bigger scale in the lower box). A fast-decaying inward current was still largely preserved after the application of DNQX and D-AP5 to block AMPA and NMDA receptors, respectively (curve 3). All responses were from the same cell and represented averages of 5 trials.

**Figure 3:**
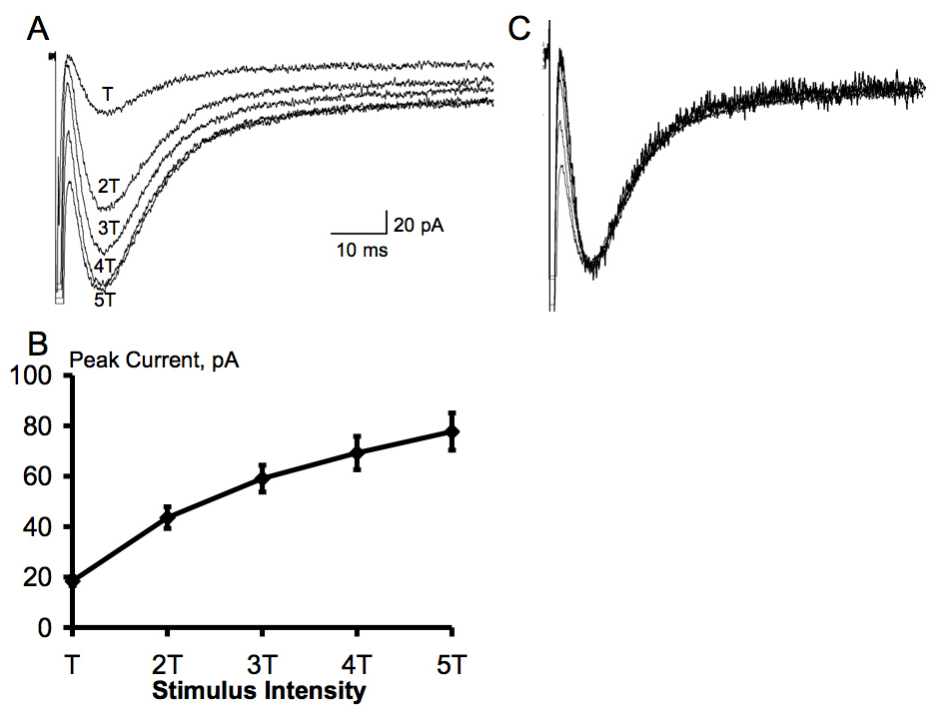
**Glutamate transporter currents recorded in spinal astrocytes reflect the intensity of synaptic activation**. A-B, the amplitude of glutamate transporter currents recorded in a spinal astrocyte increases as the intensity of dorsal root entry zone stimulus increases (n = 14). The current reaches the maximal amplitude with 4 or 5 times threshold (T). C, normalization of responses in A to the same amplitude shows the same time course of each response.

**Figure 4:**
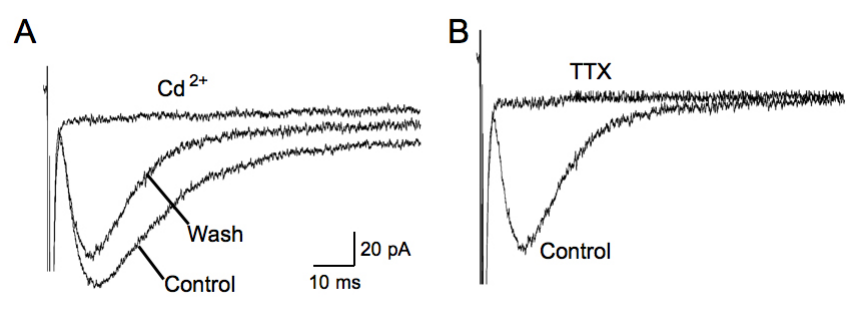
**Glutamate transporter current in spinal astrocytes is abolished by blockade of synaptic transmission**. Bath application of 40 μM CdCl2 (n = 3, A) or 1 μM TTX (n = 4, B) inhibited the evoked glutamate transporter current in spinal astrocyte, indicating the synaptic origin of these currents.

Single electrical stimuli delivered at the dorsal root entry zone evoked a large inward current in astrocytes held at a membrane potential of -80 mV (n = 15, Fig. 2). This inward current decayed slowly, often lasting over 10 seconds. In the presence of Ba^2+^, the slowly decaying inward current was blocked and a fast inward current unmasked (n = 15, Fig. 2). The fast inward currents were characterized by rapid onset with a rising τ of 3.3 ± 0.3 ms, relatively slow decay τ of 11.3 ± 0.6 ms and half decay time of 13.9 ± 0.3 ms when evoked by maximal (5 × threshold) stimuli (n = 15, Fig. 3A). Stimulus evoked inward currents in astrocytes were strongly, but reversibly, inhibited by addition of cadmium (CdCl_2, _40 μM, n = 3) or tetrodotoxin (TTX, 1 μM, n = 4) to the bath to block presynaptic calcium channels and voltage-sensitive sodium channels, respectively (Fig. 4). Thus, glutamate transporter currents elicited in response to dorsal root entry zone stimulation is dependent on the synaptic release of glutamate. The minimal intensity threshold stimulus to evoke glutamate transporter currents ranged between 0.5 and 1.0 mA. The mean amplitude of transporter currents at threshold stimulation was 18.4 ± 1.7 pA (n = 14). Increases in stimulus intensity resulted in an increase in evoked glutamate transporter current amplitude to a maximal mean response of 77.7 ± 7.4 pA (n = 14) evoked at 4 to 5 times threshold strength (Fig. 3A, B). The time course of glutamate transporter currents evoked by varying stimulus strength was assessed by scaling each of the responses to the amplitude of the maximal response. The overlay of these scaled currents shown in Fig. 3C indicates that the stronger stimuli did not change the time course of evoked glutamate transporter currents.

### Pharmacological characterization of glial glutamate transport currents evoked by dorsal root entry zone stimulation

**Figure 5:**
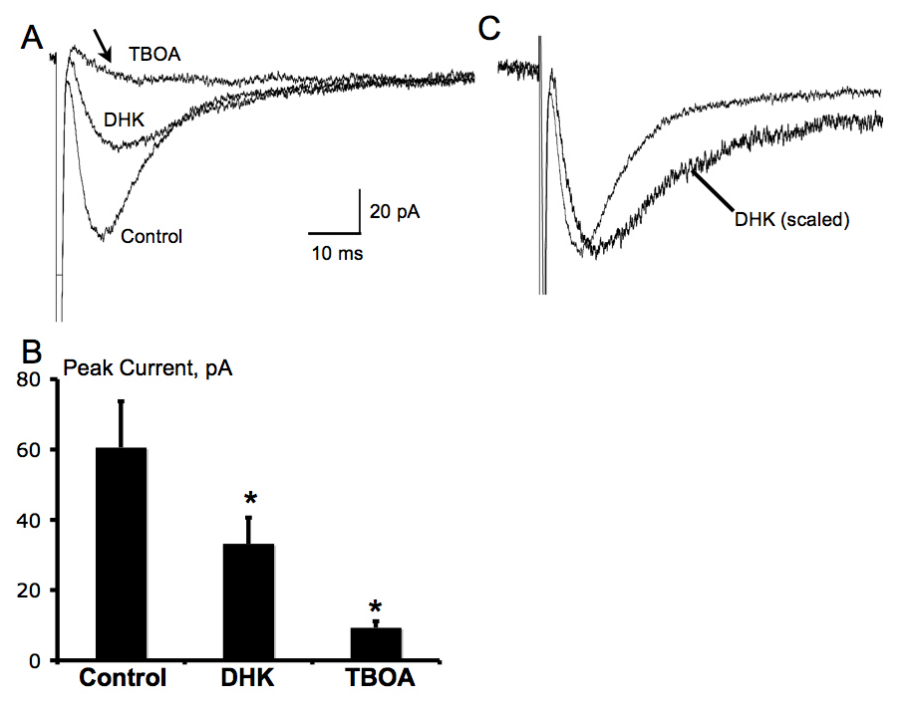
**Pharmacological characterization of glutamate transporter currents in spinal astrocytes evoked through dorsal root entry zone stimulation**. A-B, bath application of DHK (300 μM), a selective antagonist for glial GLT-1 glutamate transporter, partially blocked the evoked glutamate transporter current (n = 6), while TBOA, a non-selective antagonist for glial glutamate transporter, almost completely blocked the evoked glutamate transporter current (n = 15). Notice that a slow-decaying inward current (arrow) was not blocked by TBOA. *P < 0.01, DHK or TBOA vs. Control, Student's paired *t*-test. C, DHK slowed the decay of the glutamate transporter current, which was shown when the response in DHK was scaled to the peak of the control response (n = 6).

The fast inward current remained following addition of DNQX [6,7-Dinitroquinoxaline-2, 3-dione (10 μM)], AP-5 [D-2-Amino-5-phosphonopentanoic acid (25 μM)], bicuculline methiodide (10 μM)] and strychnine hydrochloride (5 μM)] to the bath and thus was not mediated by AMPA, NMDA, gamma-amino-butyric acid-A (GABA_A_) or glycine receptors (n = 15, Fig. 2). These currents were also insensitive to the GABA transporter inhibitor SKF-89976A (25 μM, n = 3) and nipecotic acid (100 μM, n = 3), and glycine transporter inhibitor sarcosine (500 μM, n = 3). In contrast, this current was inhibited by glutamate transporter antagonists. Application of dihydrokainate (DHK, 300 μM), a nontransported inhibitor selective for the glial GLT-1 transporter (IC_50 _= 23 μM for GLT-1 vs. = 3 mM for GLAST & EAAC1) [20], inhibited the evoked response by 45.6% ± 6.4% (n = 6; Fig. 5A, B). While DL-threo-β-benzyloxyaspartate (TBOA, 100 μM), a nonselective glial transporter inhibitor (IC_50_= 19 μM for EAAC1 [21], 68 μM for GLAST, and 6 μM for GLT-1, [22]), inhibited the current by 82.7% ± 2.1% (n = 15; Fig. 5A, B). After application of DHK, the decay of the remaining current was slowed (11.6 ± 1.4 vs. 17.9 ± 2.6 ms before and after DHK, n = 6, *P *< 0.01, Student's paired *t*-test), visibly when the response with DHK was scaled to the peak of the control response (Fig. 5C). The prolongation of the response in the presence of DHK suggests that synaptically released glutamate remains elevated in the extracellular space longer when uptake into astrocytes is reduced [17]. Blockade of glutamate transporter currents with TBOA revealed a residual small slow-decaying inward current (Fig. 5A, arrow) confirm the fast inward current (Fig. 2) as produced by glutamate transporter activity in dorsal horn astrocytes [17,23,24].

### Evoked glutamate transporter currents in astrocytes were gradually decreased in response to repetitive stimulation

**Figure 6:**
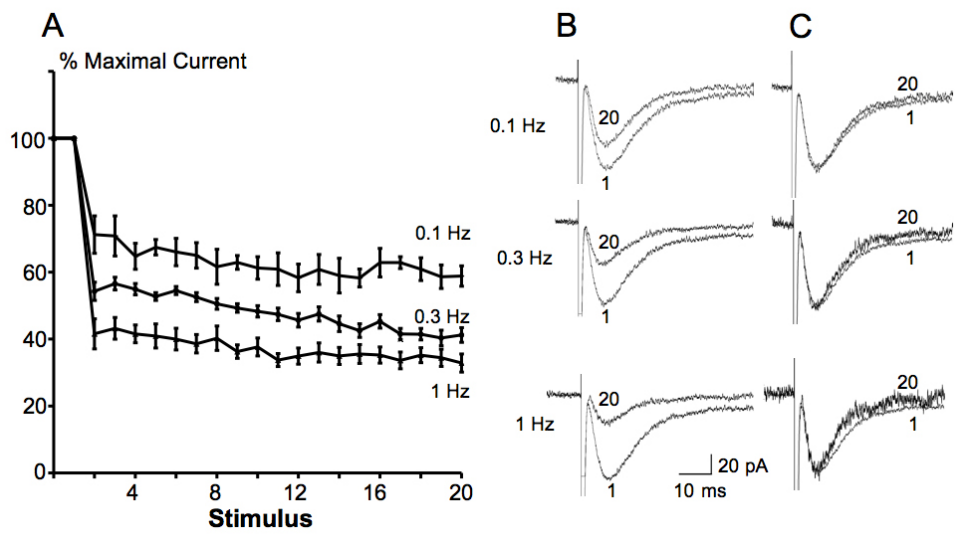
**Glutamate transporter currents in spinal astrocytes evoked by dorsal root entry zone stimulation with different frequencies**. A, glial transporter currents gradually reduced during repetitive stimulation at 3 different frequencies. Transporter currents were reduced to a greater degree with higher frequency of stimulation. B, representative responses of glial transporter currents evoked by stimulation with different frequencies, the response to the first ("1") and twentieth ("20") stimuli are displayed. All responses were from the same cell and represented averages of 5 trials. C, comparison of time courses between responses to the 1st and 20th stimuli shown in panel B, normalized to the same amplitude. Note that the response to the 20th stimulus showed similar kinetic characteristics when compared to the response to the 1st stimulus.

Repetitive dorsal root entry zone stimulation was found to result in reduced transporter currents. Stimuli adequate to evoke maximal responses (2.5 – 5 mA, 0.2 ms) were repeated at different interstimulus intervals (0.1, 0.3 and 1 Hz). As shown in Fig. 6, the evoked currents in astrocytes were significantly decreased following the second stimulus (71.2% ± 5.6% maximal current at 0.1 Hz, n = 5, *P *< 0.01, One-way ANOVA), then continued going down slowly during stimulation at all frequencies tested (58.8% ± 3.1% maximal current following the last stimulus at 0.1 Hz, n = 5). As the frequency of the stimulation went higher, the decrease in amplitudes of evoked glutamate transporter currents became greater (71.2% ± 5.6% maximal current following the second stimulus at 0.1 Hz, n = 5 vs. 41.6% ± 4.4% at 1 Hz, n = 15, *P *< 0.01, One-way ANOVA) and remained at the lower level (58.8% ± 3.1% maximal current following the last stimulus at 0.1 Hz, n = 5 vs. 32.8% ± 2.7% at 1 Hz, n = 15, *P *< 0.01, One-way ANOVA). When scaled to the peak of the maximal current, the evoked current following each stimulus had the same time course when compared to the maximal currents (Fig. 6C).

## Discussion

The electrogenic properties of glutamate uptake through glutamate transporters was used in this study to record real time physiological responses of spinal dorsal horn astrocytes to sensory inputs [6,15,25]. The results show that (1) stimulation of spinal cord dorsal root entry zone elicits glutamate transporter currents in astrocytes located in SG region, (2) spinal astrocytes express the glutamate transporters GLT-1 and GLAST, and (3) the glutamate transporter currents in spinal astrocytes share similar characteristics with that in hippocampal astrocytes and cerebellar Bergmann glia.

### Identification of spinal astrocytes

Astrocytes in spinal grey matter could be identified by their electrophysiological characterization and immunocytochemical staining for GFAP [19]. None of the recorded cells showed any Na^+ ^currents or action potentials to intracellular current injection, which differentiated them from neurons. These cells also had low input resistance and very negative resting membrane potential, characteristic of glial cells. To identify astrocytes from other types of glial cells, we first compared the pattern of membrane current responses to voltage steps. Astrocytes displayed symmetrical outward and inward currents without decay in response to a series of hyperpolarizing and depolarizing voltage steps [19]. The functional identification criteria were validated with immunostaining to GFAP, a well-known marker of astrocytes.

### Glutamate transporter uptake currents in spinal astrocytes

It has been demonstrated that glutamate transporter proteins are expressed abundantly in hippocampal astrocytes and Bergmann glial cells by physiological and molecular studies [16-18,26-30]. Spinal astrocytes in this study showed slowly decaying inward currents in response to dorsal root entry zone stimulation similar to that observed in hippocampal astrocytes in response to afferent stimulation of Shaffer collaterals [17,23,24,31]. The long-lasting inward currents in forebrain neurons were presumably due to potassium redistribution in the extracellular space and could be blocked by an ionotropic glutamate receptor antagonist, kynurenate [17,23,24,31]. Astrocytes potentially sense extracellular potassium concentration through inwardly rectifying potassium channels in their cellular membrane [32,33]. The majority of the long-lasting inward current in spinal astrocytes was confirmed as mediated by influx of potassium ions as it was blocked by Ba^2+ ^applied in the extracellular bath and resulted in an unmasking of a transient, fast inward current. Persistence of the residual rapid onset and fast-decaying inward current in the presence of antagonists to ionotropic glutamate, GABA_A _and glycine receptors and the sensitivity of this current to glutamate transporter blockers (TBOA and DHK) identifies this current as mediated by electrogenic uptake of glutamate [17,26,31]. This current could potentially be mediated by glutamate metabotropic receptors, which were not tested in this set of experiments. Yet, this possibility seems remote as the comparable fast inward current in hippocampal astrocytes was not affected by metabotropic receptor antagonists in other studies [17,18].

The majority of synapses in the CNS are ensheathed by glial processes [34], giving a structural basis of glia-neuronal communication. In the present study, the elimination of glial glutamate transporter currents in response to afferent stimulation by the blockade of presynaptic release of glutamate with TTX or Cd^2+ ^indicated that these currents were evoked by synaptic activation. The glutamate transporter current in spinal astrocytes in response to afferent stimulation had similar dynamic properties to those observed in glial cells of hippocampus and cerebellum [17,18,26,31]. The rapid onset of evoked spinal glial currents with little delay after stimulation suggests that glutamate diffuses out of the synaptic cleft rapidly in spinal dorsal horn as well, reaching glutamate transporters within a millisecond following release. Insufficient function of glutamate transporters in spinal astrocytes would therefore be expected to result in glutamate "spillover" to extrasynaptic spaces and activate high affinity receptors such as NMDA and metabotropic glutamate receptors at extrasynaptic sites and/or on neighboring synapses. This finding suggests the possibility of an entirely new mechanism of enhanced synaptic transmission in spinal cord during hyperalgesia and chronic pain. The partial inhibition of the response by DHK and almost complete inhibition by TBOA indicated the co-existence of two subtypes of glutamate transporters in spinal dorsal horn astroctyes: GLT-1 and GLAST. It is consistent with the GLT-1 and GLAST mRNA/immunohistochemical staining in rodent spinal cord at this age [12,35,36].

### Functional implication of glutamate transporters in spinal astrocytes

Although both neurons and glial cells express glutamate transporters and participate in clearing extracellular glutamate, several lines of evidence suggest that the vast majority of synaptically released glutamate is transported into glia [35,37-39]. The high-affinity glutamate transporters expressed in astrocytes are essential for taking up the majority of glutamate released at excitatory synapses, maintaining low extracellular glutamate and preventing glutamate neurotoxicity [35,37-39]. Blocking glial glutamate transporters results in both exaggerated amplitude and longer duration excitatory postsynaptic currents (EPSCs) [40-43], suggesting that glial glutamate transporters play a critical role in limiting the extrasynaptic diffusion of glutamate [44]. The loss of glial glutamate transporter GLAST or GLT-1 produced elevated extracellular glutamate levels, neurodegeneration characteristic of excitotoxicity, and a progressive paralysis [37]. Mice lacking GLT-1 show lethal spontaneous seizures and increased susceptibility to acute cortical injury due to increased level of residual glutamate in the brain [38]. Although it remains unknown how neuronal and glial glutamate transporters function differently in spinal sensory processing and chronic pain, the glial glutamate transporters certainly draw more attention since more than 80% extracellular glutamate is uptaken by glial cells. There have been several studies showing that glutamate transporters may also play a similar role in spinal cord. Immunohistochemical studies reveal that GLT-1 and GLAST are abundantly expressed in astrocytes but not neurons or microglia of spinal cord dorsal horn [14,35,36,45]. The glutamatergic synaptic transmission in spinal lamina II neurons was altered by pharmacologically blocking GLT-1 [9]. Pharmacological inhibition of glutamate transporters in the spinal cord elevates spinal extracellular glutamate concentrations and produces spontaneous nociceptive behaviors [10], and also resulted in the hypersensitivity of spinal dorsal horn neurons to mechanical and thermal stimuli [11]. Down-regulation of glutamate transporter protein expression and attenuation of glutamate uptake activity in the spinal dorsal horn is associated with neuropathic pain induced by peripheral nerve injury [12,13,45] or by chemotherapy [14]. Gene transfer of GLT-1 in to spinal astrocyes attenuated the induction but not maintenance of inflammatory and neuropathic pain in rats [46].

However, not all studies have been in agreement on the role of glial glutamte transporters in pain signaling. For example, the induction of allodynia following intrathecal injection of prostaglandin E2, prostaglandin F2, NMDA or AMPA was diminished by spinal application of TBOA [47]; and inhibition of GLT-1 activity or expression reduced formalin-induced nociceptive behavior [48]. It remains unclear whether these data indicate that glutamate transporters show plasticity with the onset of pathological pain or whether in these latter studies an excess of glutamate resulted in activation of presynaptic metabotropic receptors that in turn resulted in an anti-nociceptive response profile.

It is well known that repetitive low-frequency noxious stimuli applied to a spinal dorsal root result in a cumulative depolarization and a progressive increase in action potential output from dorsal horn neurons ("Windup") [49-51]. The cellular mechanisms responsible for the generation of wind-up are complex and still incompletely understood. Although the buildup of synaptically released glutamate was thought to be involved in cumulative depolarization of postsynaptic dorsal horn neurons with repeated stimuli, there has not been an efficient way to measure the concentration of glutamate in synaptic cleft in spinal cord. Since the synapses are ensheathed by glial processes [34], measurement of glial glutamate uptake currents induced by activation of the transporters has been suggested as an independent method of monitoring the presynaptic release of glutamate [17,26]. Several studies have shown that synaptically evoked glutamate transporter currents could be used to monitor the presynaptic release of glutamate during long-term potentiation [23,24,52]. The data shown here is consistent with this thesis as glutamate transporter currents in spinal astrocytes increase in amplitude and duration with increasing strength of single afferent volleys delivered to the dorsal root entry zone. The range of stimulus intensities delivered to the dorsal root entry zone, 0.5–5 mA in amplitude and 0.2 ms in duration, were adequate to encompass both low-threshold non-nociceptive as well as higher threshold, potentially nociceptive fibers [53,54]. The constancy of time course of glutamate transporter currents across stimulus intensities indicate that spinal astrocytes use similar transporter capacity to manage glutamate release from both sets of afferents and additionally that there is normally a reserve capacity for both types of inputs [39]. Thus, the gradual reduction in transporter currents observed with repetitive stimulation at the dorsal root entry zone across frequencies raises intriguing interpretative possibilities. At least two explanations come to mind. One, the reduced currents could reflect reduced release of presynaptic glutamate or alternatively. Two, it could reflect the saturation of the glial transporters during repetitive stimulation. If saturated, it would have been expected that later evoked transporter currents would have a longer decay time resulting from the build-up of higher concentrations of glutamate in the extrasynaptic space, but this was not observed. Moreover, glutamate transporters have very rapid turn-over rate in the continuous presence of glutamate [17,39], and the total transport capacity of glial transporters were not overwhelmed during a brief high-frequency stimulation train at near-physiological temperature, even when a majority of the transporters are blocked by a competitive antagonist [55]. Thus, it seems unlikely that the glial transporters were saturated during low-frequency repetitive dorsal root entry zone stimulation in this study. Altenratively, it might be a concern that the cotransport of 2 Na^+ ^:1Glu^- ^with countertransport of 1 K^+ ^and 1 OH^- ^involved in glutamate uptake through glutamate transporter would result in translocation of 1 net positive charge and reduce the net electric gradient. But the activation of a Cl^- ^conductance concomitant with transport would provide a potential mechanism to offset the depolarizing action of transmitter reuptake and dampen cell excitability [56]. Hence, the decrease of glial glutamate transporter currents recorded here during repetitive stimulation is concluded to reflect reduced release of presynaptic (afferent-derived) glutamate, which argues against the build-up of glutamate in the synaptic cleft in the formation of "windup" following repetitive stimulation. Further studies need to be done to clarify this issue.

## Conclusion

The present study showed that spinal dorsal horn astrocytes possess functional glutamate transporters GLT-1 and GLAST. These glutamate transporters in spinal astrocytes could be activated by synaptic transmission in spinal dorsal horn. The glutamate transporter currents in spinal astrocytes share some similar characteristics with that in hippocampal astrocytes and cerebellar Bergmann glia.

## Methods

### Animals

All experiments were approved by the Institutional Animal Care and Use Committee at the University of Texas M. D. Anderson Cancer Center and were performed in accordance with the National Institutes of Health Guidelines for Use and Care of Laboratory Animals. Male Sprague-Dawley rats (4–6 weeks; Charles River Laboratories, Wilmington, MA) were used for this study and were housed under a 12 h light/dark cycle with free access to water and food. Every effort was made to limit the numbers, pain and discomfort of animals used.

### Spinal cord slice preparation

Spinal cord slices were prepared as described previously [9,53]. Briefly, 4- to 6-week-old rats were deeply anaesthetized with isoflurane inhalation (3%). Previous studies have shown this age rat fully capable of mature nociceptive signaling and development of hyperalgesia [57]. After a laminectomy was performed, the lumber spinal cord was quickly removed and placed into ice-cold oxygenated (95%O_2 _+ 5% CO_2_) solution consisting of (mM): Sucrose, 117; KCl, 3.6; NaH_2_PO_4_, 1.2; MgCl_2_, 1.2; CaCl_2_, 2.5; NaHCO_3_, 25; Glucose, 12. The pia-arachnoid membrane was carefully peeled off and a block of the spinal cord from L3 to S1 was embedded in 4% agar. Transverse slices (400 μm thick) from lumber segments L4 to L6 were cut on a vibratome (series 1000, Technical Products International Inc., St. Louis. MO). The slices were then transferred to Kreb's solution (composition in mM: NaCl, 117; KCl, 3.6; NaH_2_PO4, 1.2; MgCl_2_, 1.2; CaCl_2_, 2.5; glucose, 11; NaHCO_3_, 25) bubbled with 95%O_2 _and 5% CO_2 _at 37°C, and allowed to equilibrate at least for 1 h before recording. The rats were euthanized by overdose of isoflurane (%5) inhalation after the spinal cord was removed.

### Electrophysiology

Whole-cell patch clamp recordings from glial cells in superficial spinal cord were obtained as previously described [19]. An individual slice was transferred to a recording chamber and continually perfused with oxygenated Kreb's solution (3 ml/min) at 37°C. Cells in the substantia gelatinosa (SG) region of spinal dorsal horn (lamina II) were visualized using a 60× water-immersion objective on an upright microscope (Olympus, BX50WI, Japan) equipped with infrared and differential interference contrast (DIC) optics. Patch pipettes were made from borosilicate glass (World Precision Instruments, Sarasota, FL) with resistances of 4–8 MΩ when filled with pipette solutions containing (in mM): 145 K-gluconate, 5 NaCl, 1 MgCl_2_, 0.2 EGTA, 10 HEPES, 2 Mg-ATP and 0.1 Na-GTP, PH 7.2. Membrane currents were measured with the patch-clamp technique on the whole-cell recording configuration [58]. Whole-currents were amplified using a Multiclamp 700B amplifier (Axon Instruments, Sunnyvale, CA), filtered at 5 kHz and sampled at 10 kHz in digital forms using a Digidata 1322A digitizing board (Axon Instruments, Sunnyvale, CA) interfaced with a computer system. Access resistance was monitored continuously throughout the experiments and was typically 10–20 MΩ. The recording was abandoned when the access resistance changed more than 20%. The input resistance (Rin) of the cell was calculated based on the steady state current change during application of a 10 mV depolarizing and/or 10 mV hyperpolarizing pulse. The membrane capacitance (Cm) of the cell was calculated from the transient capacitive currents during the application of a 10 mV depolarizing or hyperpolarizing pulse, using a single spherical compartment model. The resting membrane potential (Vm) was recorded in current clamp mode. For all the experiments, glial cells were recorded at a holding potential of -80 mV unless otherwise noted. Single stimuli of intensity 0.5–5 mA; 0.2 ms, was applied through a concentric bipolar stainless steel electrode placed in the dorsal root entry zone [9,53,54]. Evoked responses were elicited with a constant-current stimulator (Master 8, A.M.P. Instruments Ltd, Israel). Leak subtraction and compensations of capacitance and series resistance were not applied. To label cells for later morphological identification and antigenic identification, 0.1% Lucifer Yellow (dilithium salt) was added into the pipette solution. The morphological characteristics were examined with fluorescence illumination after recording. The images were captured with a digital camera and stored in digital form for later analysis.

### Immunocytochemistry

After recordings, slices were transferred to a fixation medium containing 4% paraformaldehyde in phosphate buffer (PB) and stored at 4°C. The next day, slices were washed three times with phosphate-buffered saline (PBS) for 1 h and were incubated for 15 min with 1% Triton X-100, 5% normal donkey serum (NDS, Chemicon) in PBS. After that slices were incubated for 1 h in the blocking solution containing 0.2% triton, 5% NDS in PBS. Slices were then incubated with Cy3-conjugated antibody against GFAP (Sigma, dilution on 1:500) for 2 h at room temperature in the blocking solution. Slices were then washed in PBS and mounted on glass coverslips and were viewed on a Nikon microscope (Nikon, Eclipse E600, Japan) with a X-cite 120 fluorescence illumination system (EXFO, Canada).

### Chemcials

6,7-Dinitroquinoxaline-2, 3-dione (DNQX), D-2-Amino-5-phosphonopentanoic acid (D-AP5), DL-threo-β-benzyloxyaspartate (TBOA), SKF-89976A, nipecotic acid, sarcosine and dihydrokainate (DHK) were obtained from Tocris (Missouri, USA). All other chemicals were obtained from Sigma-Aldrich (St. Louis, MO, USA) unless otherwise noted.

### Statistics

All results are presented as means ± S.E.M. The error bars in all figures indicate S.E.M. Differences between means were tested for significance using Student's paired *t*-test or One-way ANOVA followed by post hoc, pair-wise compasions (Student-Newman-Keuls method) with an alpha value of P < 0.05.

## Abbreviations

SG: substantia gelatinosa; DNQX: 6,7-Dinitroquinoxaline-2, 3-dione; D-AP5: D-2-Amino-5-phosphonopentanoic acid; TTX: tetrodotoxin; EAAT: excitatory amino acid (glutamate) transporter; AMPA: α-amino-3-hydroxyl-5-methyl-4-isoxazole-propionate; NMDA: N-methyl-D-aspartate; GABA: gamma-aminobutyric acid; TBOA: DL-threo-β-benzyloxyaspartate; DHK: dihydrokainate; GFAP: glial fibrillary acidic protein; EPSC: excitatory postsynaptic current; HEPES: 4-(2-hydroxyethyl)-1-piperazineethanesulfonic acid; EGTA: ethyleneglycol-bis (β-aminoethyl)-N, N, N', N'-tetraacetic acid; PB: phosphate buffer; NDS: normal donkey serum.

## Competing interests

The authors declare that they have no competing interests.

## Authors' contributions

HZ carried out all experiments, performed the statistical analysis, and drafted the manuscript. WX assisted with the immunocytochemical experiments. PMD contributed to the design of the study, the data analysis and interpretation, and critical review of the manuscript. All authors have read and approved the final manuscript.
